# Beyond Refractive Error: Myopia’s Exponential Burden on Retinal Health with Each Diopter

**DOI:** 10.21203/rs.3.rs-7329335/v1

**Published:** 2025-08-18

**Authors:** Leo Arnal, Yeabsira Mesfin, Christine Xu, Anish Salvi, Kapil Mishra, Chase A. Ludwig

**Affiliations:** Byers Eye Institute, Stanford University School of Medicine; University of California, San Francisco School of Medicine; Byers Eye Institute, Stanford University School of Medicine; Byers Eye Institute, Stanford University School of Medicine; University of California, Irvine School of Medicine; Byers Eye Institute, Stanford University School of Medicine

**Keywords:** myopia, retina, choroidal neovascularization, myopic macular degeneration, foveoschisis, macular hole, rhegmatogenous retinal detachment, foveal retinal detachment

## Abstract

**Background:**

As myopia reaches epidemic levels worldwide, its role in driving vision-threatening retinal complications is increasingly urgent. This study quantifies the burden of myopia by examining its association with key retinal diseases and how risk escalates with increasing severity.

**Methods:**

We conducted a retrospective cohort study using the STARR clinical data warehouse, including all patients with ≥1 documented eye visit. Myopia severity was defined by spherical equivalent and axial length, classifying patients as non-myopic, myopic, highly myopic, or severely myopic. Primary outcomes included six retinal diseases associated with myopia: choroidal neovascularization (CNV), myopic macular degeneration (MMD), foveoschisis, macular hole (MH), rhegmatogenous retinal detachment (RRD), and foveal retinal detachment (FRD). Adjusted logistic regression estimated odds by myopia severity and spherical equivalent. Mean age at diagnosis was compared across groups.

**Results:**

Retinal complications occurred at younger ages with increasing myopia severity. Compared to non-myopes, myopic, highly myopic, and severely myopic patients had 2.45 (95% CI: 2.36–2.55), 2.46 (95% CI: 2.31–2.62), and 8.15 (95% CI: 7.17–9.27) times higher odds, respectively, of developing any retinal complication. Per diopter increase in myopia, the odds of each complication increased: CNV (OR 1.11; 95% CI: 1.09–1.12), MMD (OR 1.22; 95% CI: 1.18–1.25), foveoschisis (OR 1.22; 95% CI: 1.16–1.28), MH (OR 1.06; 95% CI: 1.05–1.08), FRD (OR 1.23; 95% CI: 1.16–1.32), and RRD (OR 1.10; 95% CI: 1.10–1.11). In severe myopes, odds were markedly elevated: CNV (OR 22.90), MMD (OR 60.19), foveoschisis (OR 102.98), MH (OR 6.69), FRD (OR 22.72), and RRD (OR 6.84).

**Conclusions:**

Myopia is independently associated with higher odds of retinal diseases, and this risk increases incrementally with severity. These findings support a dose-response relationship and highlight the importance of early risk stratification, tailored monitoring, and timely referral in patients with high and severe myopia.

## Introduction

By 2050, nearly half of the global population is projected to be diagnosed with myopia (1). This surge has been attributed to growing environmental and lifestyle factors, such as an increased time spent indoors, on electronic devices, and performing near-work activities (2, 3). Clinically, the ocular complications of myopia can culminate in irreversible vision loss and significantly impair patients’ quality of life. Patients have highlighted the inconvenience of wearing glasses, blurry vision, activity limitations, and low selfesteem as among the most pertinent issues impairing their quality of life (4). Furthermore, this growing prevalence of myopia globally creates economic burdens as well. In Singapore, the average individual cost for children diagnosed with myopia is approximately $148 per year and rises to $709 per year later into adulthood (5, 6). Meanwhile, the loss of productivity due to vision loss in myopic patients has been estimated to cost countries between 6.7–9.4 billion dollars annually (7). Ultimately, the disease burden of myopia extends beyond vision impairment by reducing patients’ quality of life, increasing healthcare costs, and lowering productivity.

Axial elongation is the primary driver in the pathophysiology of myopia (8). This stretching is associated with a decrease in choroidal thickness and retinal pigment epithelium cell layer density while also promoting the development of lacquer cracks and defects in the Bruch’s membrane (8). Consequently, because axial elongation compromises the structural integrity of the retina, myopic eyes are predisposed to ocular complications. Choroidal neovascularization (CNV), myopic macular degeneration (MMD), foveoschisis, macular holes (MH), foveal retinal detachment (FRD), and rhegmatogenous retinal detachments (RRD) are among the retinal sequelae of myopia that can lead to irreversible vision loss if not managed properly (4). Growing evidence implicates dopamine pathways and the amount of time spent outdoors as critical regulators of these anatomical changes in the sclera and retina (9). For instance, dopaminergic agents have been shown to be effective in reducing axial elongation and myopic shift in in animal models (10, 11). Meanwhile, other evidence suggests that light from outdoor activities stimulates the synthesis and release of dopamine by retinal cells (9).

The surging prevalence of this refractive disorder despite its economic and disease burden highlights the importance of understanding its pathophysiology and associated complications. Every diopter increase in myopia can increase the prevalence of its complications, and so consequently, slowing its progression can lower patients’ risk of other ocular disorders (12, 13). This epidemiological study aims to contextualize the retinal sequelae of myopia. By exploring how refractive status, axial length, and spherical equivalents are associated with different ocular complications, we hope to quantify myopia’s relationship with different retinal diseases. Such findings can inspire more personalized risk stratification models and surveillance measures, encouraging better visual outcomes for myopic patients.

## Methods

The research leveraging the STARR database adhered to HIPAA regulations and received approval from the Institutional Review Board (IRB) subcommittees at Stanford Hospital. This retrospective cohort study utilized the STARR database, which contains records for over 5 million pediatric and adult patients at Stanford University dating back to 1995. The database is updated in real-time and incorporates patient data, including demographic information, clinical events, and exam findings such as, diagnoses, refractive errors, axial lengths, from Epic systems implemented at Stanford University since 2008.

We analyzed patients from a secure data repository containing all patients in STARR with at least one documented eye visit. Our primary outcomes were retinal sequelae associated with myopia— choroidal neovascularization (CNV), myopic macular degeneration (MMD), foveoschisis, macular hole (MH), rhegmatogenous retinal detachment (RRD), and foveal retinal detachment (FRD). Exposure was defined by myopia status, which was determined via spherical equivalents. Patients were classified as either non-myopes (>-0.05 D), myopes (≤-0.5 D to -6 D), high myopes (≤-6 D to -14 D), or severe myopes (≤-14 D). Race and ethnicity data were sourced from STARR. Race was categorized as Asian, American Indian, Black or African American, Native Hawaiian, White, Unknown, or Other, while ethnicity was grouped as Hispanic or Latino, Not Hispanic or Latino, or Unknown.

Prevalence tables were constructed to characterize patients without myopia, with myopia, with high myopia, and with severe myopia. These tables were stratified by age (in 10-year bins from 20 to 90+), sex, race, ethnicity, and retinal sequelae. Additionally, we compared the prevalence of the myopic groups using Chi-square tests to assess statistical differences between groups.

We also examined the mean age at diagnosis for each retinal sequela, stratified by myopia status. An ANOVA was utilized to compare the mean ages across each myopic group. A logistic regression model assessed the likelihood of each retinal sequelae, comparing each myopia group to non-myopes. Another model evaluated the association between each of the retinal sequelae and every spherical equivalent diopter increase in myopia. In all models, each retinal sequela was treated as a binary outcome, defined by the presence of the corresponding ICD-10 codes in the patient records. Repeat analysis was also performed after categorizing the myopic status of the cohort by axial lengths, where patients were classified as either non-myopes (< 24 mm), myopes (24 mm- <26.5 mm), high myopes (26.5 mm-<30 mm), and severe myopes (≥ 30 mm).

## Results

Our cohort consisted of 282,810 patients (**Table 1**). Approximately, 79.83% were diagnosed with no myopia, 15.92% with myopia, 3.72% with high myopia, and 0.51% with severe myopia. Patients with CNV, FRD, foveoschisis, MH, MMD, and RRD represented 0.14%, 0.0%, 6.0%, 0.34%, 0.01, and 1.92% of non-myopes, respectively. Meanwhile, they consisted of 0.85%, 0.01%, 0.04%, 2.18%, 0.10%, and 7.44% of myopes and 1.21%, 0.09%, 0.05%, 2.27%, 0.31, and 12.92% of high myopes. For severe myopes, they made up 5.95%, 0.42%, 0.70%, 5.67%, 1.89, and 23.44% of patients. Additionally, patients who identified as female represented 54.05%, 54.07%, 54.79%, and 47.17% of non-myopes, myopes, high myopes, and severe myopes, respectively. Patients who self-identified as white represented the largest proportion of non-myopes (45.74%) and myopes (43.82%), while those identifying as Asian consisted of 40.34% of high myopes and 35.76% of severe myopes. Meanwhile, non-Hispanic patients consisted of the greatest proportion of non-myopes (73.57%), myopes (81.35%), and severe myopes (77.75%). When comparing these myopic groups, a significant difference was observed after stratifying by age (p < 0.001), sex (p < 0.001), race (p < 0.001), ethnicity (p < 0.001), and retinal sequelae (p = 0.002). **Supplemental Table 1** contains demographic characteristics of the cohort when defining myopic status by axial length.

**Figure 1:**
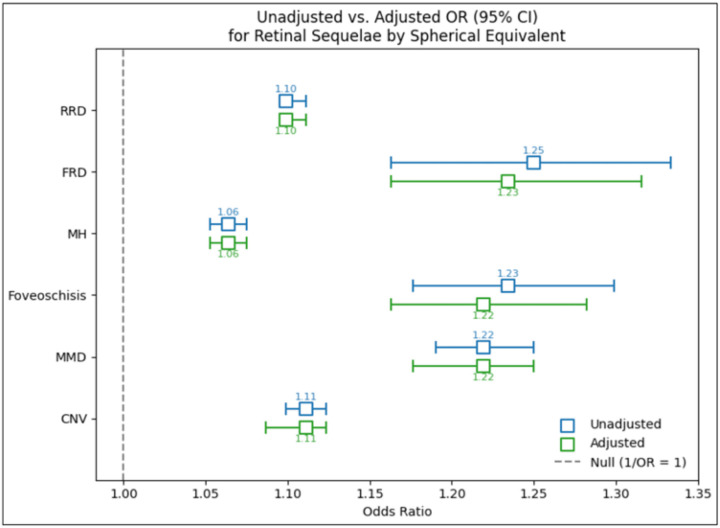
Association between myopic spherical equivalents and each retinal sequelae

The likelihood of each retinal sequelae was found to be associated with myopic status (**Table 2**). Compared to non-myopes, patients with myopia, high myopia, and severe myopia were associated with a 2.45 (95% CI: 2.36–2.55), 2.46 (95% CI: 2.31–2.62), and 8.15 (95% CI: 7.17–9.27) fold higher likelihood of being diagnosed with any of the retinal sequelae, respectively. Meanwhile, such patients were 2.49 (2.20–2.81), 1.65 (95% CI: 1.34–2.04), and 22.90 (95% CI: 17.19–30.50) times higher likelihood of being diagnosed with CNV. A diagnosis of MMD was 5.34 (95% CI: 3.70–7.71), 5.72 (95% CI: 3.48–9.42), and 60.19 (95% CI: 32.44–111.69) times more likely in myopes, high myopes, and severe myopes, respectively. A diagnosis of MH was associated with a 2.49 (95% CI: 2.31–2.70), 1.34 (95% CI: 1.16–1.55), 6.69 (95% CI: 5.14–8.70) times higher likelihood, respectively. A diagnosis of FRD was associated with a 4.72 (95% CI: 1.66–13.43), 40.30 (95% CI: 14.52–111.88), and 22.72 (95% CI: 6.23–82.88) times higher likelihood, respectively. A diagnosis of RRD was associated with a 2.24 (95% CI: 2.15–2.34), 2.66 (95% CI: 2.49–2.84), and 6.84 (95% CI: 5.94–7.87) higher likelihood, respectively. Lastly, a diagnosis of foveoschisis was associated with a 10.95 (95% CI: 5.81–20.64) and 102.98 (95% CI: 35.43–299.27) higher likelihood among myopes and severe myopes. **Supplemental Table 2** outlines these results when categorizing myopic status by axial lengths. Additionally, a significant relationship was also observed between each retinal sequelae and spherical equivalents (Fig. 1). Every diopter increase in myopia was associated with a 1.11 (95% CI: 1.09–1.12), 1.22 (95% CI: 1.18–1.25), 1.22 (95% CI: 1.16–1.28), 1.06 (95% CI: 1.05–1.08), 1.23 (95% CI: 1.16–1.32), and 1.10 (95% CI: 1.10–1.11) times higher likelihood of developing CNV, MMD, foveoschisis, MH, FRD, and RRD, respectively. **Supplemental Fig. 1** demonstrates the association between each sequela and increasing axial lengths.

**Figure 2:**
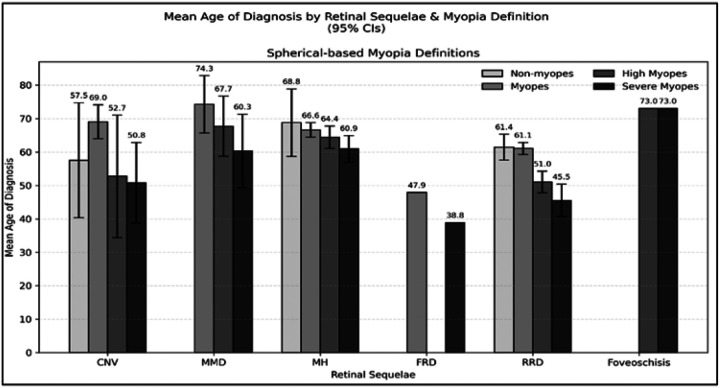
Mean age of diagnosis for retina sequelae stratified by myopia status determined by spherical equivalents

Figure 2 reports the mean age for the diagnoses of each of the retinal sequelae. On average, CNV was diagnosed at 57.5 ± 17.2, 69.0 ± 5.1, 52.7 ± 18.3, and 50.8 ± 12.1 years for non-myopes, myopes, high myopes, and severe myopes, respectively (p = 0.02). Similarly, MH at 68.8 ± 10.1, 66.6 ± 2.2, 64.4 ± 3.4, and 60.9 ± 4.0 years (p = 0.58), while RRD was diagnosed at 61.4 ± 3.9, 61.1 ± 1.8, 51.0 ± 3.2, and 45.5 ± 4.9 years (p > 0.0001), respectively. MMD was diagnosed at 74.3 ± 8.6, 67.7 ± 9.0, and 60.3 ± 11.0 years for myopes, high myopes, and severe myopes (p = 0.24). FRD was diagnosed at 47.9 ± 0.0 and 38.8 ± 0.0 years for myopes and severe myopes (p = NA), while foveoschisis was diagnosed at 73.0 ± 0.0 and 73.0 ± 0.0 years for high myopes and severe myopes (p = NA). **Supplemental Fig. 2** demonstrates these results when categorizing myopic status by axial lengths.

## Discussion

The rising prevalence of myopia globally warrants exploration of how it contributes to retinal diseases. This study demonstrated that myopia is associated with a higher likelihood of the retinal sequelae examined. By stratifying this association according to patients’ myopic status and spherical equivalent, we highlight how the strength of this predisposition towards retinal diseases increases with higher degrees of myopia as well. Patients with severe myopia were also found to be diagnosed with these retinal diseases earlier, reaffirming the positive association between the severity of myopia and the likelihood of these ocular complications.

We observed a significant difference in the prevalence of non-myopia, myopia, high myopia, and severe myopia when stratifying our cohort by age, sex, race, and ethnicity. These demographic variables are known contributors to the epidemiology of myopia. Younger age is associated with faster progression of myopia and predisposes patients to more severe manifestations in the future (14). One study found that female patients not only were 1.81 times more likely to develop myopia than males but also to progress faster, at a rate of approximately 0.02 D per year (15). Similarly, another reported Asian children to have comparable progression rates in myopia with White children, but significantly faster rates than Hispanic, Black, and Native American children (16). Our findings reaffirm these reports for a larger U.S. based population.

Myopia was also observed to increase the likelihood of CNV, MMD, foveoschisis, MH, FRD, and RRD. Axial elongation in myopic eyes induces structural changes to the retina that compromise its integrity, implicating myopia in the development of these ocular complications. Seko et al., for example, demonstrated how the stretching of the retinal pigment epithelial layer can lead to the upregulation of vascular endothelial growth factor, offering a mechanistic model for the development of CNV in myopic eyes (17). Choroidal thinning in elongated eyes has also been proposed to lead to localized ischemia with subsequent upregulation of angiogenic factors, leading to CNV as well (18). Meanwhile, axial elongation can also exert tractional forces on the retinal layers that promote the development of MH and RRD (19). The mechanical stress associated with it can also create posterior staphylomas along the posterior pole of the eye. These distortions of the scleral wall, along with the development of lacquer cracks and chorioretinal atrophy, weaken the macula and encourage foveoschisis, FRD, and MMD (4, 20). This study supports such pathophysiologic explanations by substantiating how higher degrees of myopia and longer axial lengths can incrementally increase the odds of these retinal complications, corroborating the role of myopia in each pathophysiology.

Prior studies have also echoed our results. In their systematic review, Wong et al., measured the incidence rate of CNV in eyes with pathologic myopic to be approximately 10.2% over a 10-year follow-up period (21). Our results similarly highlight the association between CNV and myopia, while also quantifying the incremental growth in the relationship with higher myopic spherical equivalents. Meanwhile, Haarman et al., similarly demonstrated how the likelihood of retinal complications increases with increasing degrees of myopia. They observed low, moderate, and high myopes to be associated with a 13.57-, 72.74-, and 845.08-fold higher likelihood of MMD and a 3.15-, 8.74-, and 12.62-fold higher likelihood of RRD, respectively (22). Lastly, Kobayashi et al., observed a positive correlation between the age of onset of MH and both the severity of myopia (R = 0.689) and axial length (R = 0.723) (23). As with these prior studies, our findings support how myopia predisposes patients to ocular complications. However, our analysis pushes further by detailing the incremental changes in these relationships with varying myopic statuses, axial lengths, and spherical equivalents.

Ultimately, as the global prevalence of myopia rises, this study offers a more nuanced understanding of the retinal complications facing myopic patients. Our findings can help inform risk stratification models when evaluating such patients, encouraging the earlier detection and management of these diseases. For instance, such knowledge can support the identification of high-risk patients in need of more regular surveillance and support more informed patient counseling. During eye examinations, it can also cue physicians and patients on which exam findings and symptoms should flag their concern for these retinal complications as well, encouraging reshaping referrals to retina specialists and improving the prognostication of pathologic myopia. With earlier detection and more proactive monitoring, the visual outcomes of myopic patients may improve.

This study is among the largest cohort studies evaluating the retinal sequelae of myopia in the United States. The STARR database contains records of over 5 million patients, which helps to increase the generalizability of our results while minimizing the effects of confounders. However, despite this, patients from marginalized communities are underrepresented within the STARR database, potentially limiting the generalizability of our results to broader populations. Lastly, patients diagnosed with high myopia, severe myopia, MMD, foveoschisis, and FRD were disproportionately underrepresented within our cohort. This may have exaggerated our findings about these retinal sequelae and may also explain the insignificant results from our logistic regression. Ultimately, future studies that circumvent these restrictions by including more representative and diverse patient cohorts can improve how well such findings can be extrapolated onto larger populations. Similarly, incorporating broader clinical data, such results from eye exams or OCT imaging, can offer a more granular understanding of myopia’s involvement in the pathophysiology of these retinal diseases.

Overall, this study offers epidemiological insights into the relationship between myopia and different retinal diseases. We demonstrated how myopia predisposes patients to CNV, MMD, foveoschisis, MH, FRD, and RRD. Furthermore, our results stratified this association by patients’ myopic status and spherical equivalents. In doing so, we hope to offer a model for risk stratification when evaluating myopic patients to improve patient prognoses. With more proactive management of these patients, we hope to improve the patient counseling and visual outcomes, reducing the disease burden of myopia.

## Supplementary Material

Supplementary Files

This is a list of supplementary files associated with this preprint. Click to download.

- SupplementalInfo.docx
- Tables.docx

## Data Availability

The datasets used and/or analysed during the current study are available from the corresponding author on reasonable request

## References

[1] Holden BA, Fricke TR, Wilson DA, Jong M, Naidoo KS, Sankaridurg P, Global prevalence of myopia and high myopia and temporal trends from 2000 through 2050. Ophthalmology. 2016;123(5):1036–42. 26875007 10.1016/j.ophtha.2016.01.006

[2] Morgan IG, French AN, Ashby RS, Guo X, Ding X, He M, The epidemics of myopia: aetiology and prevention. Progress in retinal and eye research. 2018;62:134–49. 28951126 10.1016/j.preteyeres.2017.09.004

[3] Tsai T-H, Liu Y-L, Ma I-H, Su C-C, Lin C-W, Lin LL-K, Evolution of the prevalence of myopia among Taiwanese schoolchildren: a review of survey data from 1983 through 2017. Ophthalmology. 2021;128(2):290–301. 32679159 10.1016/j.ophtha.2020.07.017

[4] Sankaridurg P, Tahhan N, Kandel H, Naduvilath T, Zou H, Frick KD, IMI impact of myopia. Investigative ophthalmology & visual science. 2021;62(5):2–. 10.1167/iovs.62.5.2 PMC8083082 33909036

[5] Lim M, Gazzard G, Sim E, Tong L, Saw S. Direct costs of myopia in Singapore. Eye. 2009;23(5):1086–9. 18670466 10.1038/eye.2008.225

[6] Zheng Y-F, Pan C-W, Chay J, Wong TY, Finkelstein E, Saw S-M. The economic cost of myopia in adults aged over 40 years in Singapore. Investigative ophthalmology & visual science. 2013;54(12):7532–7. 24159089 10.1167/iovs.13-12795

[7] Ma Y, Wen Y, Zhong H, Lin S, Liang L, Yang Y, Healthcare utilization and economic burden of myopia in urban China: a nationwide cost-of-illness study. Journal of global health. 2022;12:11003. 35356656 10.7189/jogh.12.11003 PMC8934110

[8] Jonas JB, Jonas RA, Bikbov MM, Wang YX, Panda-Jonas S. Myopia: histology, clinical features, and potential implications for the etiology of axial elongation. Progress in retinal and eye research. 2023;96:101156. 36585290 10.1016/j.preteyeres.2022.101156

[9] Zhang J, Deng G. Protective effects of increased outdoor time against myopia: a review. Journal of International Medical Research. 2020;48(3):0300060519893866. 10.1177/0300060519893866 PMC7607527 31854216

[10] Junfeng M, Shuangzhen L, Wenjuan Q, Fengyun L, Xiaoying W, Qian T. Levodopa inhibits the development of form-deprivation myopia in guinea pigs. Optometry and Vision Science. 2010;87(1):53–60. 19901858 10.1097/OPX.0b013e3181c12b3d

[11] Gao Q, Liu Q, Ma P, Zhong X, Wu J, Ge J. Effects of direct intravitreal dopamine injections on the development of lid-suture induced myopia in rabbits. Graefe’s archive for clinical and experimental ophthalmology. 2006;244(10):1329–35. 10.1007/s00417-006-0254-1 16550409

[12] Bullimore MA, Brennan NA. Myopia control: why each diopter matters. Optometry and Vision Science. 2019;96(6):463–5. 31116165 10.1097/OPX.0000000000001367

[13] Bullimore MA, Ritchey ER, Shah S, Leveziel N, Bourne RR, Flitcroft DI. The risks and benefits of myopia control. Ophthalmology. 2021;128(11):1561–79. 33961969 10.1016/j.ophtha.2021.04.032

[14] Gwiazda J, Hyman L, Dong LM, Everett D, Norton T, Kurtz D, Factors associated with high myopia after 7 years of follow-up in the Correction of Myopia Evaluation Trial (COMET) cohort. Ophthalmic epidemiology. 2007;14(4):230–7. 17896302 10.1080/01658100701486459

[15] Lee SS-Y, Lingham G, Sanfilippo PG, Hammond CJ, Saw S-M, Guggenheim JA, Incidence and progression of myopia in early adulthood. JAMA ophthalmology. 2022;140(2):162–9. 34989764 10.1001/jamaophthalmol.2021.5067 PMC8739830

[16] Jones-Jordan LA, Sinnott LT, Chu RH, Cotter SA, Kleinstein RN, Manny RE, Myopia progression as a function of sex, age, and ethnicity. Investigative ophthalmology & visual science. 2021;62(10):36–. 10.1167/iovs.62.10.36 PMC8411866 34463720

[17] Seko Y, Seko Y, Fujikura H, Pang J, Tokoro T, Shimokawa H. Induction of vascular endothelial growth factor after application of mechanical stress to retinal pigment epithelium of the rat in vitro. Investigative ophthalmology & visual science. 1999;40(13):3287–91. 10586955

[18] Wakabayashi T, Ikuno Y. Choroidal filling delay in choroidal neovascularisation due to pathological myopia. British Journal of Ophthalmology. 2010;94(5):611–5. 19846414 10.1136/bjo.2009.163535

[19] STIRPE M, MICHELS RG. Retinal detachment in highly myopic eyes due to macular holes and epiretinal traction. Retina. 1990;10(2):113–4. 2402551 10.1097/00006982-199004000-00004

[20] Takano M, Kishi S. Foveal retinoschisis and retinal detachment in severely myopic eyes with posterior staphyloma. American journal of ophthalmology. 1999;128(4):472–6. 10577588 10.1016/s0002-9394(99)00186-5

[21] Wong TY, Ferreira A, Hughes R, Carter G, Mitchell P. Epidemiology and disease burden of pathologic myopia and myopic choroidal neovascularization: an evidence-based systematic review. American journal of ophthalmology. 2014;157(1):9–25. e12. 24099276 10.1016/j.ajo.2013.08.010

[22] Haarman AE, Enthoven CA, Tideman JWL, Tedja MS, Verhoeven VJ, Klaver CC. The complications of myopia: a review and meta-analysis. Investigative ophthalmology & visual science. 2020;61(4):49–. 10.1167/iovs.61.4.49 PMC7401976 32347918

[23] Kobayashi H, Kobayashi K, Okinami S. Macular hole and myopic refraction. British Journal of Ophthalmology. 2002;86(11):1269–73. 12386087 10.1136/bjo.86.11.1269 PMC1771378

